# Metagenome-Assembled Genome of *Acidibrevibacterium fodinaquatile* FLA01 from Fumarole Sediments from the Los Azufres Geothermal Field

**DOI:** 10.1128/mra.00823-22

**Published:** 2022-10-03

**Authors:** Hermes H. Bolivar-Torres, Roberto Marín-Paredes, Cristal Ramos-Madrigal, Luis E. Servín-Garcidueñas

**Affiliations:** a Laboratorio de Microbiómica, Escuela Nacional de Estudios Superiores Unidad Morelia, UNAM, Morelia, Michoacán, México; University of Delaware

## Abstract

The Los Azufres geothermal field contains diverse microbial communities inhabiting thermal springs whose bacterial genomic diversity is being analyzed. Here, we describe a metagenome-assembled genome of *Acidibrevibacterium fodinaquatile* FLA01 obtained from fumarole sediment sequencing data. The genome contained genes for carbon fixation, osmotic shock, and heavy metal resistance.

## ANNOUNCEMENT

The genus *Acidibrevibacterium* belongs to the family *Acetobacteraceae* and was first described in samples from acid mine drainage in China (1). To date, only the genome of *Acidibrevibacterium fodinaquatile* strain G45-3 is available in the GenBank database. Here, we report a draft genome sequence of *Acidibrevibacterium fodinaquatile* FLA01 obtained from a sediment metagenome.

A 20-gram fumarole sediment sample (56°C pH 3.8) was collected at the Los Azufres geothermal field in Western Mexico (19.7816° N 100.6577° W) in April of 2019. Sediment was collected at a maximum depth of 0.5 cm and stored in sterile 50 mL Falcon tubes. DNA extraction was performed using the DNeasyPowerSoil Pro kit (Qiagen) following the manufacturer's instructions. A library was prepared using the TruSeq Nano DNA (350) prep kit (Illumina). The library was sequenced producing paired-end 250-bp reads on an Illumina HiSeq2500 instrument (Macrogen Co., Seoul, South Korea). Reads were filtered for quality (>Q30) with Trimmomatic v.0.39 (2). Reads were iteratively mapped against the *A. fodinaquatile* G45-3 genome (GenBank GCA_003352165.1) using bowtie2 v.2.3.4.3 (3). Mapped reads were assembled *de novo* with SPAdes v.3.15.2 (4). Contigs contamination was assessed using CheckM v.1.0.13 (5). Scaffolding was performed with the MeDuSa v.1.6 web server (http://combo.dbe.unifi.it/medusa) (6). Gap filling was done with GMcloser v.1.6.2 (7) and sealer v.2.1.5 (8). The final assembly was reviewed with Quast v.5.0.2 (9). All tools were run with default parameters. MAG annotation was performed using the NCBI Prokaryotic Genome Annotation Pipeline (PGAP) v.5.2 (10). The 16S rRNA sequence similarity, average nucleotide identity (ANI), and digital DNA-DNA hybridization (dDDH) values were calculated using BLAST v.2.13.0 local service (11), JspeciesWS (https://jspecies.ribohost.com/jspeciesws/#analyse) (12), and the Genome-to-Genome Distance Calculator (GGDC) v.3.0 (https://ggdc.dsmz.de/ggdc.php) (13), respectively.

A 3,760,223-bp draft MAG comprising 452 contigs (*N*_50_ 62,809 bp), with a GC content of 65.5%, and genome coverage of 33×, was obtained. After scaffolding, the MAG consists of 57 scaffolds containing 3,779,387-bp (*N*_50_ 3,603,386). The MAG was estimated to be 99.25% complete, with no detected contamination. MAG annotation predicted 3,786 genes, some of them related to heat and osmotic shock, phosphate transport, and several ABC transporters. In addition, genes predicted to be involved in carbon fixation, sulfur metabolism, and heavy metal resistance were detected. Compared to the *A. fodinaquatile* G45-3 genome, 16S rRNA sequence similarity (100%), ANI (98.43% %), and dDDH (87.30%) values (14) indicate that the recovered MAG belongs to the species *A. fodinaquatile.* This study allowed the identification of the genus *Acidibrevibacterium* in the Los Azufres geothermal field. The obtained MAG may be useful for comparative analyses.

### Data availability.

The MAG of *Acidibrevibacterium* sp. FLA01 and its annotation are available in NCBI GenBank under accession numbers GCA_020418435 and JAIZPH000000000. The MAG raw reads are available in NCBI Sequence Read Archive (SRA) under accession number SRR15652788. Metagenomic sequencing data are available in SRA under accession number SRR17356570 (BioProject number PRJNA486381).
